# Liver Size in Saudi Children and Adolescents

**DOI:** 10.4103/1319-3767.45052

**Published:** 2009-01

**Authors:** Mohammad I. El Mouzan, Abdulla A. Al Salloum, Abdulla S. Al Herbish, Mansour M. Qurachi, Ahmad A. Al Omar

**Affiliations:** 1 Department of Pediatrics, King Saud University, Riyadh; 2 Department of Pediatrics, Al Yamama Hospital. Riyadh; 3 The Children's Hospital, King Saud Medical Complex, Riyadh

**Keywords:** Children, liver size, liver span, Saudi Arabia

## Abstract

**Background/Aim:**

To examine the liver size in Saudi children and adolescents.

**Methods:**

A large sample of children was selected from the general population by multistage random probability sampling for the assessment of physical growth. A random subsample of children–newborns to 18 years old–was taken from this larger sample for this study. Liver size below the costal margin and liver span along the midclavicular line were determined by physicians. Data were analyzed using SPSS software and medians and standard deviations were calculated.

**Results:**

Between 2004 and 2005, 18 112 healthy children up to 18 years of age were examined. All were term and appropriate for gestational age. There were 9 130 boys and 8 982 girls, yielding a nearly 1:1 male to female ratio. The maximum palpable liver size below the costal margin was 2.4 cm. The median and + 2 SD liver span at birth were 4 and 6.9 cm, respectively. There was no difference in the liver span between boys and girls of up to 60 months of age. Thereafter, a difference could be seen increasing with age, with girls having smaller liver spans than boys.

**Conclusion:**

This manuscript reports the liver size in Saudi children and adolescents. The data should help physicians in the interpretation of liver size determined by physical examination of children and adolescents.

The liver, like other organs of the body, grows with age. It has been estimated that between birth and adulthood, there is an at least ten fold increase in liver mass.[1] Assessment of liver size is an important part of clinical examination, and information about the normal size of the liver at various ages is necessary for the detection of hepatomegaly, a condition that needs further evaluation.[2,3] Literature reports on liver size in children are either quite old or deal with a limited number of children, and most were based on Western populations. To our knowledge, there are no data on the liver size in Arab, in general, and Saudi children and adolescents, in particular. Therefore, in this paper, we report the liver size in a large number of healthy Saudi children and adolescents.

## PATIENTS AND METHODS

This study was part of the Health Profile of Children and Adolescent Project in Saudi Arabia, designed primarily for the establishment of growth charts for Saudi children and adolescents. The detailed methodology has been reported elsewhere.[4] In brief, the original main sample (42,000) was determined by multistage probability sampling of the population from each of the 13 regions of the Kingdom, consisting of newborns and children aged up to 18 years. A subsample (18,112) was randomly selected from this population for the assessment of liver size. Only Saudi children who were term and appropriate for gestational age, with no past or present history of any symptoms or signs of liver disease, were eligible for the measurement of liver size. All measurements were performed by primary care physicians trained in the technique and provided with written instructions. The physicians had completed workshop training conducted by the investigators in each region, including theoretical explanation and practical demonstration of the method of measurement of liver size.

Inter- and intraobserver measurements were performed in about 1% of the examined children. Examination of the liver was performed as part of the general clinical examination during a house visit. The children were examined in the supine position. All physicians used a standardized technique (percussion and palpation of the upper and lower borders respectively).[5] The lower border is gently palpated in an upward direction, starting from the right iliac fossa. As soon as the edge of the liver is felt; its position is marked with a transverse line on the skin. The upper border is percussed in a downward direction and marked at the middle of the finger when a distinct change of tone to dullness is noted. Then, the examiner measures the distance between the two borders (liver span), and the distance of the lower edge from the right costal margin (liver size below the costal margin) with a stretched metric paper tape. All measurements were taken along the mid-clavicular line. Inter- and intraobserver variations were determined in 1% of the children throughout the data collection, and a difference in liver size of >1 cm led to a repetition of measurement and subsequent correction or deletion of the re-measurement.

The data were tabulated on Statistical Package for Social Sciences (SPSS) spreadsheets, which in addition to liver size data, included the age, gender, weight, and length/height of the children. The following four variables were assessed to determine the level of association with liver span: age, length/height, weight, and body surface area. While viewing the data as scatter graphs, it was found that the variance in liver span increased with each of the four variables. Therefore, the natural log of liver span was used and the variance was found to be very similar across all levels. In addition, because there appeared to be differences between boys and girls, sex-specific curves were investigated. Linear, quadratic, and cubic curves were fitted, and it was found that the latter two equations tended to have a higher r-square than a linear equation. The r-square terms for the quadratic and cubic curves for the four variables were similar, ranging from 0.383 to 0.419. Therefore, it was decided to use the natural log of liver span by age, partially because age had the higher r-squared value, and also, for ease of use by clinicians. To compute + 2 and - 2 standard deviations (SDs), the squared root of the mean square error was used, multiplied by + 2 or - 2, and then added to the constant beta term. It was found that the difference between males and females < 60 months of age was < 0.1 cm. Hence, it was decided to have unisex liver span curves for children up to 60 months of age and sex-specific curves for children aged 60 months and older.

## RESULTS

Twenty-five primary care physician members of the field teams, covering all 13 regions of the country, participated in this study. A total of 18,112 children and adolescents were examined over a period of two years (2004–2005). The children ranged from newborns to those 18 years of age. All were term and appropriate for gestational age. There were 9,130 boys and 8,982 girls, indicating a nearly 1:1 male to female ratio. Data for the liver size below the costal margin are depicted in Table 1, 2.4 cm being the maximal palpable liver size below the costal margin.

**Table 1:** Liver size (cm) below the costal margin

Age (years)	Liver size—boys		Liver size—girls	
	Median	Median + 2SD	*Median	Median + 2SD
Birth	0.6	2.4	0.4	1.6
5	0.3	1.5	0.2	1.4
12	0.1	1.0	0.1	0.9
18	0.02	0.26	0.07	0.7

* Standard deviation

**Figure 1:**
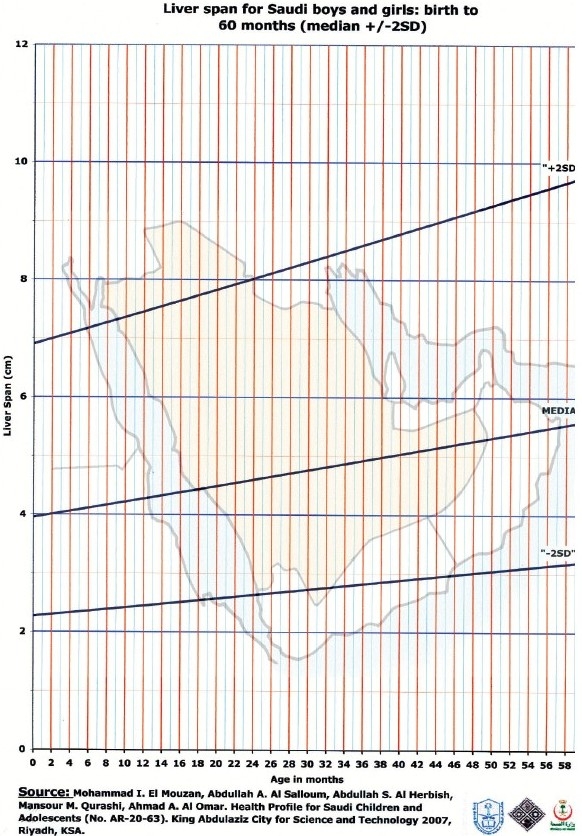
Liver span by age (0–60 months): Median ± 2SD

**Figure 2:**
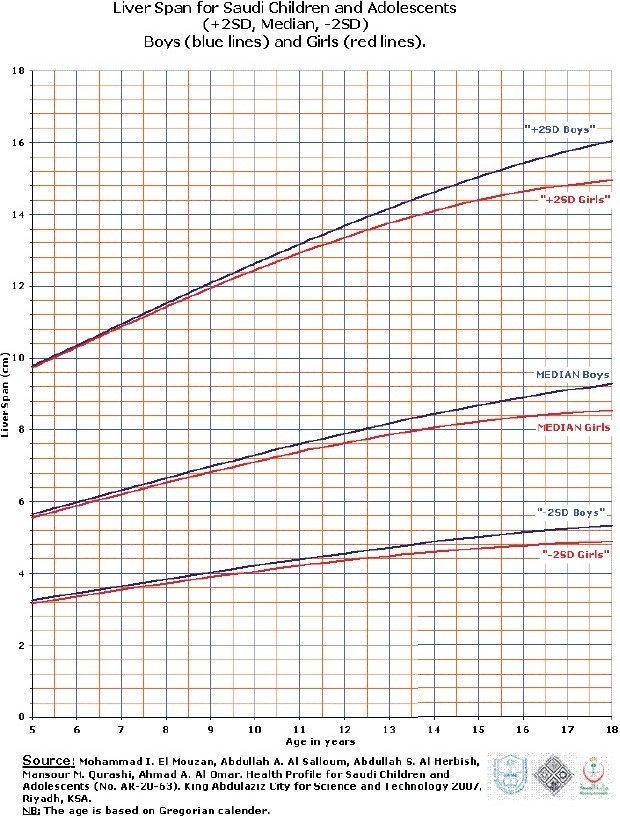
Liver span by age (5–18 years)

Liver span data are shown in Tables 2 and 3; Figure 1 shows the graphic representation of liver span for infants up to 60 months of age. At birth, the median and +2 SD liver spans were 4 and 6.9 cm, respectively. Thereafter, the span increases slowly but steadily. This pattern is similar for boys and girls. Figure 2 depicts the liver span in children and adolescents aged 5–18 years, indicating a pattern of difference in liver span between boys and girls. The girls have smaller liver span than boys, and this difference increases steadily with age.

**Table 2:** Liver span data set for children aged 0–60 months

Age (months)	+ 2 SD	*Median	- 2 SD
0	6.9	4.0	2.3
6	7.2	4.1	2.5
12	7.5	4.3	2.5
18	7.7	4.4	2.5
24	8.0	4.6	2.6
30	8.3	4.8	2.7
36	8.6	4.9	2.8
42	9.0	5.1	2.9
48	9.2	5.3	3.0
54	9.5	5.4	3.1
60	9.8	5.6	3.2

* Standard deviation

**Table 3:** Liver span data set (cm) for children and adolescents aged 5–18 years

Age	+2 SD*	Median	-2SD	+2 SD	Median	-2 SD
5	9.8	5.6	3.3	9.7	5.6	3.2
6	10.4	6.0	3.5	10.3	5.9	3.4
7	10.9	6.3	3.7	10.9	6.2	3.6
8	11.5	6.7	3.8	11.4	6.5	3.7
9	12.1	7.0	4.0	12.0	6.8	3.9
10	12.6	7.3	4.2	12.5	7.1	4.1
11	13.2	7.6	4.4	13.0	7.4	4.2
12	13.7	7.9	4.6	13.5	7.6	4.4
13	14.2	8.2	4.7	13.8	7.9	4.5
14	14.6	8.5	4.9	14.1	8.1	4.6
15	15.0	9.0	5.0	14.4	8.2	4.7
16	15.4	8.9	5.1	14.6	8.4	4.8
17	15.8	9.1	5.3	14.8	8.5	4.8
18	16.0	9.3	5.4	14.9	8.5	4.9

* Standard deviation

## DISCUSSION

The clinical assessment of liver size remains an important part of physical examination and knowledge of its normal values at different ages is essential in children and adolescents. Such a procedure is usually the first step in detecting an abnormal size of the liver. Although there are more accurate methods of assessing liver size such as ultrasonography,[6] such methods are rarely available in clinics and primary health care centers, and are usually considered as a second step. Indeed, recent editions of major pediatric textbooks[2,3] as well as current clinical teaching still emphasize the value of determining the liver size as an important aspect of the clinical skills of medical students and physicians. The determination of the liver span is essential whenever the size of the liver edge below the costal margin is bigger than expected. Accordingly, knowledge of the normal range of liver size in children of all ages is still needed by physicians in day-to-day clinical practice.

The present study is part of a national project to establish reference values of some health indices (e.g., growth and blood pressure) in children and adolescents. These data on liver size are the largest and most recent that have been reported to date, and establish the liver size in normal Saudi children and adolescents aged up to 18 years. The technique adopted in this study—percussion of the upper border and palpation of the lower border—avoids any interference from gas in the bowel and therefore, is considered one of the most sensitive techniques that correlates well with ultrasonography.[6,7]

The liver size below the costal margin (BCM): In this study, the liver size below the costal margin showed the expected gradual decrease with advancing age. In newborns, the liver size BCM was higher in boys than in girls, but the values became similar thereafter. The upper limit of the size of liver edge BCM was 2.3 cm in the newborn, is within the reported normal range of 1.6–4.4 cm, but clearly below the upper limit.[8] However, this finding is consistent with the normal size reported in pediatric textbooks, one of which considers a figure of 1–3 cm to be normal for the first six months of life, without providing any information about medians and standard deviations.[9]

The liver span: It is generally accepted that liver span is a better estimate of liver size than the size BCM for all ages.[10,11] In this study, the lack of any difference in liver spans between boys and girls of up to 60 months of age, and the larger liver span in boys thereafter, justified separate liver span curves for these two age groups. Thus, our findings are similar to others’ findings.[2,11]

In this report, newborns had a median and upper limits of normal liver span (mean + 2SD) similar to those found in reports from the USA[12] and China.[7] Data on the liver span in older children are both scarce and quite old. Lawson *et al*.[11] reported the mean liver span in 350 children, including newborns to those aged up to 20 years. Their study sample consisted of both healthy and children with minor illnesses attending the Children's Hospital Medical Center. While the general shape and differences between boys and girls were found to be similar, the means in our study are consistently higher across all age groups by 0.5–1 cm. However, the mean liver span in our study is 1–2 cm lower than that reported in an older study by Younoszai *et al*.[13] Again, their study used a different technique (percussion of the lower border) than ours in a group of children aged from 5 to 13 years. It is difficult to compare the results of the above studies with these in the present report because of differences in the type of samples that are more selective, smaller in size, and much older than ours (more than a difference of 20 years). In addition, there are no more recent data on the liver span in older children to allow for proper comparison. Accordingly, the difference between our results and those in available literature[11,13] may be due to sampling, variation between generations, or to ethnic variation between populations.

Finally, it is necessary to highlight the limitations of this study. The most important one is that our clinical findings were not supported by more accurate measurements such as ultrasonography. Therefore, this report should be considered a clinical estimation rather than the establishment of true liver size. The issue of subjectivity of measurements is minimized by inter- and intraobserver verification as well as by the large number of cases.

In conclusion, the present study reports the estimation of liver size in a large sample of healthy Saudi children and adolescents. It is hoped that these data will help physicians in the interpretation of liver size values that have been found on physical examination.
